# Consensus study on the health system and patient-related barriers for lung cancer management in South Africa

**DOI:** 10.1371/journal.pone.0246716

**Published:** 2021-02-11

**Authors:** Witness Mapanga, Shane A. Norris, Wenlong C. Chen, Charmaine Blanchard, Anita Graham, Laurel Baldwin-Ragaven, Tom Boyles, Bernard Donde, Linda Greef, Ken Huddle, Busisiwe Khumalo, Elizabeth Leepile, Buhle Lubuzo, Raynolda Makhutle, Yusuf Mayet, Merika Tsitsi, Preethi Mistri, Keletso Mmoledi, Mpho Ratshikana-Moloko, Rajen Morer, Lauren Pretorius, Jayshina Punwasi, Guy A. Richards, Paul Ruff, Dineo Shabalala, Maureen Sibadela, Nita Soma, Michelle Wong, Maureen Joffe

**Affiliations:** 1 Division of the Wits Health Consortium, Non-Communicable Diseases Research (NCDR), Faculty of Health Sciences, University of the Witwatersrand, Johannesburg, South Africa; 2 Division of Medical Oncology, Department of Internal Medicine, Faculty of Health Sciences, University of the Witwatersrand, Johannesburg, South Africa; 3 SAMRC/Wits Developmental Pathways to Health Research Unit, Department of Paediatrics, Faculty of Health Sciences, University of the Witwatersrand, Johannesburg, South Africa; 4 National Cancer Registry, National Health Laboratory Service, Johannesburg, South Africa; 5 Sydney Brenner Institute for Molecular Bioscience, Faculty of Health Sciences, University of the Witwatersrand, Johannesburg, South Africa; 6 Centre for Palliative Care, Faculty of Health Sciences, University of Witwatersrand, Johannesburg, South Africa; 7 Division of Pulmonology, Department of Internal Medicine, Faculty of Health Sciences, University of the Witwatersrand, Johannesburg, South Africa; 8 Department of Family Medicine and Primary Care, School of Clinical Medicine, Faculty of Health Sciences, University of the Witwatersrand, Johannesburg, South Africa; 9 Helen Joseph Hospital, Auckland Park, Johannesburg, Gauteng, South Africa; 10 ANOVA Health Institute, Johannesburg, South Africa; 11 London School of Hygiene and Tropical Medicine, London, United Kingdom; 12 Division Radiation Oncology, Department of Radiation Sciences, Faculty of Health Sciences, University of the Witwatersrand, Johannesburg, South Africa; 13 The Cancer Alliance, Johannesburg, South Africa; 14 Gauteng Department of Health, Tladi Provincial Clinic, Johannesburg, South Africa; 15 Discipline of Public Health Medicine, School of Nursing and Public Health, University of the KwaZulu Natal, Durban, South Africa; 16 Chris Hani Baragwanath Academic Hospital, Johannesburg, South Africa; 17 CANSA Association South Africa, Johannesburg, South Africa; 18 Campaigning for Cancer, Johannesburg, South Africa; 19 Alpha World Ministries Social Care Centre, Johannesburg, South Africa; King’s College London, UNITED KINGDOM

## Abstract

**Background:**

Lung cancer is the highest incident cancer globally and is associated with significant morbidity and mortality particularly if identified at a late stage. Poor patient outcomes in low- and middle-income countries (LMIC’s) might reflect contextual patient and health system constraints at multiple levels, that act as barriers to prevention, disease recognition, diagnosis, and treatment. Lung cancer screening, even for high-risk patients, is not available in the public health sector in South Africa (SA), where the current HIV and tuberculosis (TB) epidemics often take precedence. Yet, there has been no formal assessment of the individual and health-system related barriers that may delay patients with lung cancer from seeking and accessing help within the public health care system and receiving the appropriate and effective diagnosis and treatment. This study aimed to derive consensus from health-system stakeholders in the urban Gauteng Province of SA on the most important challenges faced by the health services and patients in achieving optimum lung cancer management and to identify potential solutions.

**Methods:**

The study was undertaken among 27 participant stakeholders representing clinical managers, clinicians, opinion leaders from the public health sector and non-governmental organisation (NGO) representatives. The study compromised two components: consensus and engagement. For the consensus component, the Delphi Technique was employed with open-ended questions and item ranking from five rounds of consensus-seeking, to achieve collective agreement on the most important challenges faced by patients and the health services in achieving optimal lung cancer management. For the engagement component, the Nominal Group Technique was used to articulate ideas and reach an agreement on the group’s recommendations for solution strategies and approaches.

**Results:**

Public health sector stakeholders suggested that a lack of knowledge and awareness of lung cancer, and the apparent stigma associated with the disease and its risk factors, as well as symptoms and signs, are critical to treatment delay. Furthermore, delays in up-referral of patients with suspected lung cancer from district health care level were attributed to inadequate knowledge arising from a lack of in-service training of nurses and doctors regarding oncologic symptoms, risk factors, need for further investigation, interpretation of x-rays and available treatments. At a tertiary level, participants suggested that insufficient availability of specialised diagnostic resources (imaging, cytological and pathological services including biomolecular assessment of lung cancer), theatres, cardiothoracic surgeons, and appropriate therapeutic modalities (chemotherapeutic agents and radiation oncology) are the main barriers to the provision of optimal care. It was suggested that a primary prevention programme initiated by the government that involves private-public partnerships may improve lung cancer management nationally.

**Conclusions:**

Considerable barriers to the early identification and treatment of lung cancer exist. Finding solutions to overcome both individual and health-system level obstacles to lung cancer screening and management are vital to facilitate early identification and treatment, and to improve survival. Furthermore, research on inexpensive biomarkers for asymptomatic disease detection, the introduction of diagnostic imaging tools that utilise artificial intelligence to compensate for inadequate human resources and improving clinical integration across all levels of the healthcare system are essential.

## Background

Lung cancer is the most frequently diagnosed cancer, the leading cause of cancer deaths in males and the second leading cause in females globally [1, 2]. In 2018, some 2,093,876 new cases of lung cancer and 1,761,007 deaths occurred globally which constituted close to 1 in 5 (18.4%) all cancer deaths [3]. By 2030, in low-to-middle-income countries (LMICs), the mortality of cancers of the trachea, bronchus, and lung are projected to be ranked sixth after cardiovascular diseases, HIV/AIDS, chronic obstructive pulmonary disease (COPD) and lower respiratory tract infections [4].

The most important cause of lung cancer is tobacco smoking [5], which although decreasing in high-income countries (HIC’s) is increasing in LMICs including South Africa (SA) [6, 7]. Lung cancer morbidity and mortality trends thus vary widely across regions, reflecting variations in tobacco use [6]. Other predisposing factors for lung cancer include older age, genetic susceptibility, occupational and environmental exposures (asbestos, silica, and radon gas from uranium mining), indoor biomass-fuel exposure, outdoor pollution, and chronic inflammatory lung diseases–in particular, tuberculosis (TB) and HIV [1, 5, 8–13], all risks that are endemic in Southern Africa.

The World Health Organisation (WHO) recommends a comprehensive approach to lung cancer prevention, control, and management, including increased smoking cessation initiatives among current smokers and eliminating smoking initiation. In addition, the WHO recommends that screening for lung cancer should be implemented in line with effective treatments for reducing morbidity and mortality [14]. Such treatments include surgery for the earlier-stage disease, radiotherapy, chemotherapy, and various palliative and supportive procedures to reduce morbidity and mortality and improve quality of life [14].

However, evidence has shown that the availability and utilisation of lung cancer screening services are generally inadequate in LMICs [15]. Due to the nonspecific and delayed manifestation of symptoms in lung cancer, most patients tend to seek care and be diagnosed at a late stage of the disease, which results in dismal health outcomes [14]. In many settings, barriers both individual and at the level of the health system have been reported to pose challenges to patients seeking and accessing health care services. These include a lack of patient knowledge about the risks for and symptoms of lung cancer, the significant financial costs for health services, sociodemographic characteristics linked to poverty, myths or inaccurate beliefs about lung cancer screening or diagnostic workup, distrust of the medical system, and the inconveniences of screening processes and receipt of care [15]. Within the health-system, insufficient knowledge of lung cancer, poor recognition of early symptoms confounded by other more common respiratory diseases, and long queues in primary care clinics, delay decision-making by front line health care workers (HCW) and prevent timeous referral of patients, [15, 16]. Insufficient, and at times absent, diagnostic imaging and pathology services within secondary/district hospital services also contribute to delayed diagnosis and treatment.

In SA, though significantly under-reported by the pathology-based SA National Cancer Registry [17], lung cancer is the third most common cancer in males and fifth most common in females with 1,791 males (age-standardised incidence rate (ASR) per 100,000 of 10.12 and 936 females (ASR/100,000 of 3.95). The Public Health Sector treats around 80% of the SA population. Most patients are from socioeconomically disadvantaged communities and smoking prevalence is high (29% of males and 7.3% of females [18]. Almost all patients present with advanced-stage lung cancer to primary care clinics where they routinely undergo chest X-rays, HIV–testing and sputum and gene-Xpert tests for tuberculosis diagnostic workup. Patients with negative TB results, and those with unresolved symptoms after TB treatment, are referred either to secondary/district or tertiary hospital respiratory clinics for an investigation to obtain tissue for cytological or histopathological diagnosis workup. In the Public Health Sector, treatments are provided by tertiary academic hospitals. Respectable cases (less than 2%) receive surgery performed by cardiothoracic surgeons but those that are deemed unfit for surgery will receive palliative chemotherapy and or radiation treatments at mainly urban-based tertiary/quaternary hospitals located in approximately 10 major cities of SA.

There is a dearth of local-research-based knowledge in SA, on the individual and health-system related barriers that delay access to the public health system and consequently to diagnosis. Understanding the individual and health-system related barriers to early detection and effective management may assist in the development and testing of feasible and affordable patient and health system interventions. To begin addressing this gap, this study aimed to: (i) derive consensus from health-system stakeholders on the most important challenges faced by the health services and patients in achieving optimal lung cancer management in the urban Gauteng Province of South Africa; and (ii) formulate potential solutions to these challenges and in so doing identify critical research needs.

This study was conducted in Johannesburg, in the Gauteng Province of SA. Gauteng is the most populous of the nine provinces, with an estimated population of around 15 million (25.8% of the total population), of whom around 11 million reside in urban Johannesburg. Economically, Gauteng is the financial and manufacturing hub of the sub-Saharan African region and in 2016, it contributed almost 35% to SA’s Gross Domestic Product (GDP). SA has endemic HIV and tuberculosis (TB); the HIV prevalence is approximately 15% for adults [17], and one of the highest TB incidences in the world (>500/100,000) of whom 60% have HIV [19].

## Methodology

### Study protocol

The study compromised two components: consensus and engagement. For the consensus component, the Delphi Technique [20, 21] was employed with open-ended questions and item ranking to achieve collective agreement on the most important challenges faced by patients and the health services in achieving optimal lung cancer management in the setting. The results of the consensus component were shared with the stakeholders in an engagement workshop to obtain input on solutions to the challenges that were identified and to investigate research opportunities. For the engagement component, the Nominal Group Technique [22, 23] was used to articulate ideas and reach an agreement on the groups’ recommendations for solution strategies and approaches. Ethical approval to conduct the study was obtained from the University of the Witwatersrand Human Research Ethics Committee (Medical) (Clearance Certificate #M171027 dated 27 October 2017).

### Stakeholder recruitment

The majority (around 65%) of South Africa’s (SA) 59 million population reside in urban metropolitan centres, and the 80% socioeconomically disadvantaged population accesses the resource-constrained SA Public Health System. Our study setting is the city of Johannesburg (~12 million residents) within the most populated SA Province, Gauteng. Johannesburg is home to around 20 high volume primary care Community Health Centres (CHC) that receive patients with respiratory symptoms for a chest x-ray and blood diagnostic workup, referred from many small primary health clinics. Patients with persistent lung symptoms following tuberculosis screening are referred to around 10 district secondary hospitals for diagnostic workup including CT scanning who in turn refer patients to 3 tertiary hospitals affiliated with the University of the Witwatersrand for further bronchoscopy investigation and treatments. Most tertiary academic hospitals are in urban cities and are affiliated with University medical schools. Our study findings pertain to the majority urban metropolitan centres of South Africa and barriers to care identified are anticipated to be exacerbated in rural settings where patients travel long distances to healthcare facilities with fewer resources.

As summarised in Table 1, we undertook a comprehensive scoping of 97 specialist and general practitioner clinicians, nurses, facility and provincial managers and nongovernment organisation stakeholder experts, all of whom have been working in respiratory and cancer care within the primary, secondary or tertiary tiers of Public Health services for a minimum of 5 years. The majority of the 27 stakeholders that consented and participated in the study have served for more than 20 years within the SA Public Health services. Stakeholder experts were initially contacted by telephone and email and invited to participate. The first Delphi round was surveyed through anonymised email responses and the subsequent rounds were conducted at a workshop in Johannesburg with anonymous electronic voting by participants.

**Table 1:** Summary study setting and recruitment approach.

South Africa, population 2019	~59 million of whom 65% reside within urban settings
Gauteng Province population, mainly urban	~15 million (26%)–most populous province of South Africa
Participating city, Johannesburg which includes Soweto	Population ~12 million
Tertiary Academic Hospital participant recruitment centres in Johannesburg (3)	University of Witwatersrand affiliated Charlotte Maxeke Johannesburg Academic Hospital, Chris Hani Baragwanath Academic Hospital (Soweto), Helen Joseph Hospital.
Tertiary clinicians (oncologists, pulmonologists, thoracic surgeons, pathologists, radiologists) and oncology nurses	26 approached of whom 9 consented and participated
Tertiary hospital clinical managers	6 approached of whom 3 consented and participated
Secondary level district referral hospital Respiratory Clinic Department Heads and nurses	15 from all 15 referring hospitals were approached of whom 4 consented and participated
Provincial Department Managers	10 approached of whom 2 consented and participated
Highest volume primary care facilities selected– 9 of 11 facilities approached	18 medical offices and nurses approached of whom 4 consented and participated
Nongovernmental organisation representatives supporting cancer patients	12 major NGO representatives with >5-year service experience within Johannesburg regions were approached of whom 5 consented and participated
Summary participant representation- 24 in total	12 tertiary level, 4 secondary levels, 4 primary care level, 2 provincial-level managers, 5 NGO representatives

### Delphi round 1

For the first round, each participant was sent a personalised electronic information sheet that introduced the Delphi technique. The participant was then asked to generate written responses to three questions about the most important factors affecting lung cancer management in the province of Gauteng. The questions solicited participant opinions and perceptions as to which were the most important:

- *personal barriers*, *hurdles or problems that cause lung cancer patients from Gauteng communities to delay accessing and seeking help within the public health system*.
- *problems and challenges in the primary and district public healthcare referral network that cause delays in the recognition of patients with potential lung cancer and subsequent referral to district and tertiary respiratory clinics for management*.
- *barriers to effective diagnosis and treatment of lung cancer patients in Gauteng tertiary hospitals*.

For each question, participants were invited to provide at least five but no more than ten responses. These responses were not ranked in the first round. The research team derived common themes from the responses to each question, which were carried over to round 2 for ranking and consensus. Each consenting participant was assigned a study code to ensure the anonymity of responses.

### Delphi rounds 2–5

All participants from round 1 were invited to attend a workshop in-person where they went through a process of deliberation, ranking, and agreement by consensus on the top five most important themes for each of the three questions above. The voting process was facilitated through several rounds of anonymous real-time electronic surveys until statistical consensus was obtained. At the end of each round, the results for each question were presented to participants and instructions given for the next round.

For round 2, participants were asked to select and vote for the top 10 most important themes (unranked) for each question generated in round 1. The top 10 themes, plus those with tied mean scores, were carried through to the next round of voting.

In the third Delphi round, participants were asked to select and vote for the top 5 most important themes (unranked) from each question and these (including tied mean scores) were carried through to the next round.

In the fourth round of voting, the participants were asked to rank the themes of each question in order of importance. After the results from this round were presented to the participants, the floor was opened for participants to discuss and motivate for any change in theme ranking. Following this, in round 5, the participants were asked to re-rank the themes for each question. The results were then presented to the group and any change of ranking order from round 4 was discussed. The consensus was then obtained on the final rank order for each theme for each question. This concluded the Delphi survey component.

The Delphi scoring was conducted anonymously for each round of scoring. The scores were not presented at an individual level but rather presented as aggregated means for each question. Participants’ identity or position or dominant was not featured in the scoring process. Every participants’ scores were weighted equally in the aggregation of the mean.

With regards to the feedback and discussion between rounds, each participant who wished to raise a discussion, whether for or against a certain scoring, was given equal opportunity to do so. The feedback discussion process was mediated by the Delphi Host to mitigate the effects of dominant voices with perceived authority to influence discussions. Each disputed question was debated robustly.

### Delphi analysis approach

The most important ranked themes (5) for all participants were given a weighting score of 1, which decreased by an interval of 0.2, such that the least important themes (1) got a weighting of 0.2. Thereafter, the mean (average) score was calculated with a standard deviation for each ranked theme for each of the three questions. The formula for the mean weighted score was the sum of the weighted score divided by the number of votes for each theme. The theme with the highest weighted score was ranked the most important for the given question. In the event of tied weighted scores, the theme with the lowest standard deviation was ranked higher (reflecting greater consensus). The same rank-ordering process of themes was undertaken for each of the three questions posed.

### Nominal group solution-seeking approach

Drawing upon the results that emerged from the Delphi exercise reflecting the most important challenges faced by patients and the primary, secondary and tertiary health services in managing lung cancer in our urban Johannesburg setting, we used a nominal group approach to formulate potential solutions. The Nominal group technique (NGT) is defined as a structured method for group brainstorming that encourages contributions from everyone and facilitates quick agreement on the relative importance of issues, problems, or solutions. Team members begin by writing down their ideas, then selecting which idea they feel is best. During the nominal group discussion, participants were afforded a silent period where participants were asked to generate ideas. Individual ideas were then verbalised, captured verbatim on a board followed by a facilitated discussion, consolidation of each idea followed by agreement on the best ideas raised and the research opportunities.

## Results

### Process of the Delphi survey

Twenty-seven participants, with representation from each stakeholder group, took part in all rounds of the Delphi consensus process and the subsequent engagement workshop.

### Round 1

In round 1 of the Delphi survey, each participant provided electronic responses to the three questions posed. These unranked responses from the 27 participants were filtered for overlap and repetition and consolidated into a list of unique themes for each question: 33 different themes were identified for question one (Table 2); 25 for question two (Table 3); and 21 for question three (Table 4).

**Table 2:** Delphi round 1: Unranked consensus opinions on the most important problems patients with lung cancer face in seeking help and accessing the Gauteng public health system.

Rank	Consensus answers (in no order of importance)
	Lack of knowledge and awareness of lung cancer and its risk factors, symptoms and signs and thus do not take them seriously and not aware of the dangers of delay
	Poor nutrition
	Lack of smoking cessation clinics
	Most T1 lesions are asymptomatic- so when the first symptoms appear the cancer is too often advanced
	Patients live in environments where everyone smokes, and lung conditions deteriorate
	Cultural barriers and belief systems
	Preference to seek help from traditional healers or over the counter options
	Lack of media awareness and emphasis on lung cancer
	Fear -of diagnosis and treatments, stigma, the unknown or being away from family members; fear to notify their families they have cancer or being judged because they smoke; fear they may be asked to stop smoking
	Lack of caregiver to look after family members at home
	Ignorance about how to seek healthcare
	Ignorance of treatments available for lung cancer to improve QoL
	Stigma linked to misinformation- cultural factors may also cause stigma, think the disease is witchcraft
	Nearest facilities are too distant for easy access
	Long waiting times in clinics
	Costs of medical treatments
	Lack of funds to get to facilities (transport costs)
	Being turned away from clinics because arrive late due to long walking distances
	Struggle with day-to-day personal responsibilities, needs, day-to-day survival priorities, put others needs before their own healthcare needs- cause delays
	The economic impact of taking time off from work to attend primary care services
	Lack of needed caregiver to accompany patients to facilities
	Repeated visits for misdiagnoses for TB-patients lose faith in the health system and go to GPs
	Failure to come back for follow up diagnostic or treatment appointments
	Patients change their mobile numbers and then cannot be contacted or may not answer their phones from unidentified callers-fearing debt collection
	Patients endure bureaucracy at health care facilities ID, proof of residence, articulation of chief complaint
	Language barriers between patients and healthcare practitioners and thus difficult communications and understanding of doctor information
	The stigma associated with a symptom patient thinks maybe TB
	A belief that treatment is painful
	Guilt and feeling ashamed that because they smoke, they deserve cancer and maybe ostracized or ashamed to face caregivers
	Inadequate clinical knowledge of community workers for correct community messaging and symptom recognition for fast-track referrals
	Delays due to cultural beliefs where seek alternative therapies from traditional healers or over the counter options
	Patients need extra support in making them understand the diagnosis without making them feel threatened by death
	Home oxygen is not easily resourced

**Table 3:** Delphi round 1: Unranked challenges and barriers in the primary and secondary services that cause delays in the referral of patients with lung cancer to tertiary respiratory centres for diagnosis and management.

Rank	Consensus answers (in no order of importance)
	Inadequate transport e.g., ambulances, buses to ferry patients to and from facilities
	Long delays to get appointments, long waiting periods in clinics and long queues for high patient volumes and for diagnostic tests compounded by early closing times
	Primary health care is nurse-driven, and doctor supported–lung cancer not prioritized as a diagnosis-and not listed in the index of disease conditions
	Misdiagnoses linked with superficial examinations–over-emphasis on more common HIV and TB, pneumonia with a low index of suspicion for lung cancer
	Delays in getting diagnostic workup test results for imaging, cytology, pathology, and surgery
	Unwillingness for healthcare workers to consider a cancer diagnosis because of the inability to break bad news and/or accompany the patient through the journey of care
	Administration hassles—no referral forms, lack of hospital transport for referrals, obtaining informed consent, booking appointments for referrals
	Patient health awareness messaging within primary resources is not structured and sustained with no CHC outreach to the community
	Insufficient information on the prevalence of lung cancer and how best to manage it
	Using sputum only to diagnose cancer
	Biological specimens e.g., pleural fluid not sent for analysis
	Inadequate knowledge and in-service training of nurses and doctors regarding oncologic symptoms, risk factors, needs for further investigation, interpretation of x-rays and treatments available
	Too few doctors in primary and specialists employed in secondary care
	Poor communication and cooperation between primary, secondary and tertiary services–no specialist outreach and guidance to support PHC practitioners -hence bottlenecks in the referral network
	Work overload and burnout-and high staff turnover -not enough resources for high patient loads
	Substandard or absent diagnostic facilities (cytology, pathology, imaging) at secondary hospitals and X-ray facilities at primary care clinics
	Communication by practitioners with patients and caregivers is inadequate and insensitive and patients too often not informed that cancer is suspected
	No follow up of patients by the same doctors
	Lack of distress recognition and psychosocial support services for patients
	Lack of availability of treatments due to stock-outs
	Unwelcoming, demotivated uncommitted staff who turn patients away
	Nihilistic attitude to lung cancer treatment
	No systems to track and follow up patients who miss appointments even when histology clearly indicates a cancer
	Lack of guidelines and protocols for screening, referral, and management
	No time or belief in counselling for tobacco cessation

**Table 4:** Delphi round 1: Unranked challenges and barriers within the tertiary hospital respiratory services that prevent timely, accurate and effective diagnosis and treatment of patients with lung cancer.

Rank	Consensus answers (in no order of importance)
	Tertiary centres are daunting and far from patients’ homes
	Inadequate specialist diagnostic resources, services, theatres, equipment and supplies (imaging, cytology, pathology, cardiothoracic surgery, chemo, and radiation oncology) and inadequate biomolecular assessment of lung cancer
	Lack of patient navigators to navigate within tertiary hospitals
	Lack of follow up of patients and outstanding test results
	Tertiary clinics will not accept patients referred from secondary without baseline diagnostic workup which causes big delays
	Long waiting lists and delays for all diagnostic and interventional services and investigational results
	Lung cancer is difficult to diagnose properly-and distinguish primary from secondary cancers
	Lack of guidelines for referral and management of patients from primary, to secondary and tertiary clinics
	Patients are seen by unsupervised junior staff who miss diagnoses- and patients not followed up by the same doctor and poor clinical note-taking and clinical history taking
	Poor collaboration, communication, and feedback from specialists to referring doctors and within the referral network and vice versa
	Supply chain problems for drugs and treatments–stock outages, non-availability of oxygen therapy for home use
	Uncoordinated services and poor cooperation between specialist diagnostic services
	Lack of access to newer drugs which cost too much and technologies to optimally treat lung cancer–abandonment of older cheaper medicines by Pharmaceutical companies
	Late presentation of patients
	Therapeutic nihilism by Health care professions for lung cancer
	Lack of supportive and palliative care services and hospices for terminally ill patients and their families
	Absence of multidisciplinary clinics for the management of lung cancer
	The reluctance of physicians to offer recommended radical treatments such as concurrent chemo-radiation because of concerns about toxicity for patients with many comorbid conditions that they believe are unfit for treatments or delay cancer treatments
	Lost specimens, test results, and scans further delay diagnoses
	Lack of communication between doctors and patients- language and cultural barriers
	Staff burnout and disinterest

### Analysis of important problems and challenges in lung cancer management (Delphi Rounds 4–5)

Following Rounds 2 and 3 to reach consensus on the top ten and top five most important themes (unranked) respectively for each of the three questions, the voting proceeded in Rounds 4 and 5 to achieve the rank order of importance. We did not use statistics to identify ranking. Following the first Delphi round, subsequent rounds were conducted at the workshop within real-time presentations of findings to the participants. We thus calculated the mean, standard deviation (SD) and coefficient of variation for the scoring of each question and the scores were presented to the participants, discussed, and voted on for each round. The prior defined threshold for consensus was selected as 95% of those voting participants agreeing with the ranking.

### Question 1: What are the most important personal barriers, hurdles or problems that cause lung cancer patients from Gauteng communities to delay accessing and seeking help within the public health system?

Stakeholders ranked six themes as important. Among these the most important [*lack of knowledge and awareness of lung cancer and its risk factors*, *symptoms and signs that patients thus do not take seriously and are therefore not aware of the dangers of delaying seeking care*] had a mean weighted score of 0.76±0.33, whilst the least ranked theme scored 0.35±0.29 (see S1 Fig). The agreed top 5 rankings from the fourth round were then carried into the fifth and final round to be ranked/re-ranked.

In the fifth and final round, the participants re-ranked the six important themes from round 4 after motivating for a change of rankings. This process resulted in five themes being selected, with the highest-ranked having a mean weighted score of 0.84±0.32 and the least a score of 0.39±0.23 (see S2 Fig). These five themes emerged as the most important personal barriers, hurdles or problems that are likely to delay patient access to and seeking help from the public health system:

(1a) a lack of knowledge and awareness of lung cancer and its risk factors, symptoms and signs make patients unaware of the dangers of delay in seeking treatment and care–*Mean weighted score (0*.*84±0*.*32)*

(1b) repeated visits for and misdiagnosis of tuberculosis in the public primary care facilities cause patients to lose faith in the health system and seek care from private general and other practitioners—*Mean weighted score (0*.*69±0*.*27)*

(1c) patient fears of diagnosis and treatments, stigma, the unknown or being away from family members; fear to notify their families they have cancer or being judged because they smoke; fear they may be asked to stop smoking–*Mean weighted score (0*.*44±0*.*27)*

(1d) day-to-day struggles with personal responsibilities, needs and survival priorities force patients to put others’ needs before their own need to seek health care–*Mean weighted score (0*.*39±0*.*23)*

(1e) most T1 (early stage) lesions are asymptomatic, so when symptoms first appear the cancer is too often advanced–*Mean weighted score (0*.*44±0*.*28)*

### Question 2: What are the most important primary and district public healthcare referral network problems and challenges that cause delays in the recognition of patients with potential lung cancer and their referral to district and tertiary respiratory clinics for the management?

The participating stakeholders ranked five themes as important in the fourth round of the Delphi process. Among these the highest ranked theme [*inadequate knowledge and in-service training of nurses and doctors regarding oncologic symptoms*, *risk factors*, *need for further investigation*, *interpretation of x-rays and available treatments*] had a mean weighted score of 0.77±0.24. The lowest-ranked theme [*substandard or absent diagnostic facilities (cytology*, *pathology*, *imaging) at secondary hospitals and X-ray facilities at primary care clinics*] scored 0.39±0.29 (see S3 Fig). These selected and ranked themes were agreed on and carried into the fifth and final round to be discussed, ranked/re-ranked.

In the fifth and final round, following discussion, the participants were asked to re-rank the five important themes from round four. This process resulted in a mean weighted score of 0.77±0.30 for the highest-ranked theme of question two and a score of 0.34±0.27 for the least (see S4 Fig). These were the five themes that emerged as the most important challenges and barriers in the primary and secondary services that cause delays in the referral of patients with lung cancer to tertiary respiratory centres for diagnosis and management:

(2a) inadequate knowledge and insufficient in-service training of nurses and doctors regarding oncologic symptoms, risk factors, indications for further investigation, interpretation of x-rays and available treatments–*Mean weighted score (0*.*75±0*.*31)*

(2b) lack of guidelines and protocols for screening, referral, and management of patients–*Mean weighted score (0*.*77±0*.*30)*

(2c) substandard or absent diagnostic facilities (cytology, pathology, imaging) at secondary hospitals and X-ray facilities at primary care clinics–*Mean weighted score (0*.*34±0*.*27)*

(2d) delays to get appointments and diagnostic tests, long waiting periods in clinics and long queues due to high patient volumes, compounded by early facility closing times–*Mean weighted score (0*.*48±0*.*23)*

(2e) no tools or systems to track and follow up patients who miss appointments even when the diagnostic histology indicates cancer–*Mean weighted score (0*.*45±0*.*26)*

### Question 3: What are the most important barriers to effective diagnosis and treatment of lung cancer patients in Gauteng tertiary hospitals?

The participating stakeholders ranked five themes as important in the fourth round of the Delphi process. Among the themes, the one considered to be most important [insufficient availability of specialised diagnostic resources (imaging, cytological and pathological services including biomolecular assessment of lung cancer), theatres and cardiothoracic surgeons availability of therapeutic modalities such as chemotherapeutic agents and radiation oncology] had a mean weighted score of 0.76±0.24, whilst the least important[*long waiting lists and delays for all diagnostic and interventional services and investigational results*] scored 0.50±0.31 (see S5 Fig).

In the fifth and final round, following discussion, the participants were asked to re-rank the five selected themes from round four. This process resulted in the highest-ranked theme of question three to have a mean weighted score of 0.87±0.24 and the lowest a score of 0.35±0.21 (see S6 Fig). These were the five themes that emerged as the most important barriers to effective diagnosis and treatment at a tertiary level:

(3a) inadequate specialist diagnostic resources, services, theatres, equipment, and supplies (imaging, cytology, pathology, cardiothoracic surgery, chemo, and radiation oncology) and inadequate biomolecular assessment of lung cancer–*Mean weighted score (0*.*87±0*.*24)*

(3b) late-stage presentation of patients at which point very little can be done for them–*Mean weighted score (0*.*70±0*.*28)*

(3c) long waiting lists and delays for all diagnostic and interventional services and investigational results—*Mean weighted score (0*.*35±0*.*21)*

(3d) absence of multidisciplinary clinics for the management of lung cancer–*Mean weighted score (0*.*51±0*.*26)*

(3e) lack of a patient-centric approach for disease assessment and management–*Mean weighted score (0*.*39±0*.*30)*

### Solutions proposed and discussed during the nominal group engagement process

Table 5 summarizes the five key patient barriers identified by the stakeholder group and solutions suggested to address these challenges. As lung cancer symptoms are nonspecific and manifest at late-stage disease, community smoking cessation and advocacy programs against high risk occupational and environmental pollutants, were deemed to be of utmost importance. Patient lung awareness and education programs around symptoms, diagnosis and treatments and addressing fears and myths and stigmas were all deemed necessary. Multi-stakeholder partnerships and forums to strengthen lung cancer management were also suggested.

**Table 5:** Solutions suggested for the key barriers patients experience in seeking help and accessing the Gauteng health system.

Barrier Theme (Q1)	Proposed solutions
Lack of knowledge and awareness of lung cancer risk factors and symptoms	Implement community programs educating on awareness of risks and symptoms of lung cancer; patients healthcare rights and dispelling myths associated with diagnosis and treatments
Patient fears of diagnosis, treatments and stigma associated with smoking and cancer	Define and standardize appropriate attitudes and behaviours of health care workers across all levels when faced with patients at high risk for lung cancer
Most T1 (early stage) lesions are asymptomatic, so when symptoms first appear the cancer is too often advanced.	Initiate lung cancer primary prevention interventions that include community and clinic smoking cessation programs, advocacy programs to influence government policy and industry standards to limit high risk occupational environmental exposures
Patients face day-to-day struggles with personal responsibilities, needs and survival priorities and others’ need before their own need to seek health care	
Repeated visits for and misdiagnosis of tuberculosis in the public primary care facilities cause patients to lose faith in the health system	Initiate multi-stakeholder partnerships and a forum for fostering healthcare worker, NGO, fieldworker, and public health department partnerships to improve lung cancer management.

The primary clinics and secondary hospitals are faced with overwhelming challenges in identifying and fast-tracking patients at high risk for lung cancer among the large volumes of patients with HIV and TB that they must diagnose and treat. The group discussed various strategies that may strengthen the multi-tier health services involved in lung cancer management. Table 6 summarizes the stakeholder group solutions proffered for the five key barriers that emerged from the Delphi process, targeting the development of multidisciplinary teams, management guidelines, tracking tools and healthcare worker training. Essential but affordable chemotherapy drugs must be made available on hospital essential drug lists.

**Table 6:** Solutions suggested for the key challenges identified for primary and secondary facility settings that result in delays in disease recognition, diagnostic workup and management.

Barrier Theme (Q2)	Proposed solutions
Delays getting appointments and diagnostic tests, long waiting periods in clinics and long queues due to high patient volumes	Foster multidisciplinary partnerships for better integration of HIV, TB, and lung cancer diagnostic processes and prioritized referral of high-risk patients.
	Foster better coordination between clinical and diagnostic services and monitoring of delays and bottlenecks
Lack of guidelines and protocols for screening, referral, management of patients	• Develop guidelines and implementation algorithms and protocols for identifying high-risk symptomatic patients, preliminary diagnostic workup procedures, prioritized referral, and definitive diagnosis.
	• Optimum but affordable lung cancer curative and palliative drugs must be represented on hospital essential drug lists
No tools or systems to track and follow up patients who miss appointments even when the diagnostic histology indicates cancer	Develop tools and systems to track and follow up patients who miss appointments even when the diagnostic histology indicates cancer
Inadequate knowledge and insufficient in-service training of nurses and doctors	Implement ongoing in-service training of primary healthcare workers in lung cancer, guidelines, and algorithms for better management of lung cancer patients
Substandard or absent diagnostic facilities (cytology, pathology, imaging) at secondary hospitals and X-ray facilities at primary care clinics	Map the primary-secondary-tertiary health service facilities, infrastructure, equipment, processes, and services to identify gaps, bottlenecks, and opportunities for intervention.

Table 7 summarises the five key challenges for the tertiary services agreed to by the stakeholder group in the Delphi consensus and multidisciplinary interventions suggested by the nominal stakeholder group. It was proffered that nationwide smoking prevention and cessation programs be intensified and drugs and agents to assist smoking cessation to be made available on essential drug lists. Clinicians in tertiary services should lead multidisciplinary teams in all phases of patient-centred pathways to address all bottlenecks and deficiencies in lung cancer management, and electronic tracking tools be developed and implemented to track each aspect of the patient management pathway.

**Table 7:** Solution seeking themes for the key barriers identified to optimum diagnosis and treatment of lung cancer patients in Gauteng tertiary hospitals.

Barrier Theme (Q2)	Proposed solutions
• Inadequate specialist resources for imaging, cytology, pathology including molecular biomarker assessments, cardiothoracic surgery, chemotherapy and radiation oncology.	Initiate multi-disciplinary team approaches for patient-centred pathways to management
• Absence of multidisciplinary clinics for the management of lung cancer	
Late-stage presentation of patients at which point very little can be done for them	Intensify nationwide smoking prevention and cessation programs and make drugs to assist smoking cessation freely available on essential drug lists
Long waiting lists and delays for all diagnostic and interventional services and investigational results	Analysis of trends in bottlenecks, delays, stage at diagnosis, adherence to standardized guidelines and protocols.
	Develop electronic IT systems to facilitate patient navigation and track referred patients, non-compliant patients, outstanding diagnostic results, outstanding interventions
Lack of patient-centric approach for disease assessment and management	Patients that test negative for TB and/or who have unresolved symptoms following initiation of TB treatment must be fast-tracked for lung cancer assessment

### Research intervention suggested

From the discussions, it emerged that in-depth interviews with patients need to be undertaken to unpack their challenges and needs experienced in accessing and navigating the health services for their lung cancer management

## Discussion and key suggestions

The Delphi consensus and engagement with a nominal group of stakeholders involved in lung cancer management in the Gauteng province identified fifteen key themes on barriers and challenges that lung cancer patients face in accessing the public health services and the challenges and barriers experienced by clinicians and nurses in primary, secondary, and tertiary facilities in providing quality care for these patients.

Concerning patient barriers to seeking care, lack of knowledge and awareness of lung cancer and its risk factors, symptoms, and signs, was the most important factor preventing timeous access to the public health system, consistent with global findings [24–26]. On the other hand, symptoms for most lung cancer lesions manifest at advanced stages of the disease. This highlights the need to strengthen smoking prevention and cessation programs in schools, communities and clinics and provide drugs to aid smoking cessation on essential drug lists. There is also an urgent need to develop an inexpensive diagnostic biomarker for early disease detection through screening of high-risk patients. The second key barrier that emerged was the loss of faith in and distrust of the health system due to repeated fruitless visits in which lung cancer was misdiagnosed as TB [15]. This distrust requires stakeholders and the government to intervene. Through discussion, several small but tangible interventions were suggested that could be employed to regain trust. These include i) linking the TB and lung cancer units to minimize misdiagnoses and unnecessary delays in referral; ii) staff training on diagnostic indications and interpret diagnostic tests; iii) standardisation of diagnostic, referral and treatment protocols and improving channels of communication and linkages among health workers across the three levels of care within the health system.

Smokers who develop the disease face a great deal of stigma and blame and feel shame and guilt associated with smoking [27, 28] though about 15% of lung cancer patients are non-smokers [29]. Besides delay in seeking treatment and care, lung cancer patients are likely to struggle with their day-to-day personal responsibilities, needs, and priorities and this will further deter them from accessing health care on time resulting in significant negative consequences.

Key challenges and barriers in the primary and secondary health services that cause delays in the referral of patients with lung cancer to tertiary respiratory centres for diagnosis and management include inadequate knowledge about lung cancer symptoms, risk factors, indications for further investigation, interpretation of x-rays and available treatments. Doctors and nurses faced with a TB epidemic have a low index of suspicion for diagnosing lung cancer, which they misdiagnose as TB and delay referring patients for proper management [30]. These various challenges and barriers are congruent with some of the evidence from sub-Saharan Africa where the inability to access surgical care, cost of oncological care, lack of cancer specialists and poor infrastructure, were all attributed to poor prognosis among cancer patients [31, 32]. Lack of guidelines and protocols for screening, referral, and management and substandard or absent diagnostic facilities (CT imaging, cytology, pathology) at secondary hospitals and poor X-ray facilities at primary care clinics, have all contributed to the inadequate management of lung cancer patients. This has also been supported with evidence from other studies where lack of diagnostic and treatment facilities has been found to have contributed to poor management of lung cancer patients [33, 34].

Even when patients with suspicious lesions are identified and referred timeously to tertiary hospital specialist respiratory services inadequately resourced diagnostic services lead to missed and delayed diagnosis, which can result in medicolegal implications in some cases [35]. A patient-centric multidisciplinary team approach to patient management is lacking in the Gauteng public health services [36, 37]. These have proven effective in improving survival in first world settings as they foster adherence to standardized treatment guidelines and care [38–41]. Affordable chemotherapy agents must also be made available on all essential drug lists.

The solutions proposed will inform both research and implementation science agendas. This health system strengthening research is currently being implemented in our urban Johannesburg setting to promote early detection and referral of patients at their earliest entry point at primary health clinics routinely screened for tuberculosis (TB), employing trained fieldworkers to follow up patients for persistent symptoms following TB-positive treatments and navigation of symptomatic patients to secondary and tertiary levels of care for diagnostic workup. We also plan to carry out qualitative research to better understand patient and health service provider perspectives on barriers and enablers to early detection and timely referral of patients with persistent respiratory symptoms and for the integration of screening for respiratory symptoms into the routine TB screening programmes. We anticipate this kind of qualitative research will facilitate intervention development using the intervention mapping framework. In conclusion, this consensus process was valuable in bringing to the fore needs to support a solid research agenda in LMIC settings such as South Africa.

## Limitations

Since the Delphi round 1 was an idea generation stage, the research team could not gather ideas outside the approached participants. Hence this might have been a limitation of this first process because other people outside of the study might have raised important additional themes. Another limitation is that what is being reported in this paper are healthcare professionals’ interpretation of what they perceive to be patient’s concerns. We might have inferred some of these issues incorrectly without patient representation. Therefore, future research should include patient representatives.

## Conclusions

The combination of Delphi for consensus and nominal group technique for quick solution-formulation is an effective approach that enabled a multidisciplinary stakeholder group to frame the important barriers and potential solutions to lung cancer management in the South African Gauteng Province. The findings provide a roadmap for designing, implementing, and evaluating interventions to address these barriers, which are vital to improving the quality of life and survival outcomes of lung cancer patients.

## Supporting information

S1 Fig Delphi round 4: Patient barriers to access health services mean weighted scores. (TIF)

S2 Fig Delphi round 5: Patient barriers to access health services mean weighted scores. (TIF)

S3 Fig Delphi round 4: Challenges faced by the primary and district public healthcare referral facilities mean weighted scores. (TIF)

S4 Fig Delphi round 5: Challenges faced by the primary and district public healthcare referral facilities mean weighted scores. (TIF)

S5 Fig Delphi round 4: Tertiary health service barriers mean weighted scores. (TIF)

S6 Fig Delphi round 5: Tertiary health Service barriers mean weighted scores. (TIF)
