# Supplementary figures and images for: Consensus study on the health system and patient-related barriers for lung cancer management in South Africa
Source: PLoS One. 2021 Feb 11;16(2):e0246716. doi: 10.1371/journal.pone.0246716 (PMC7877667; doi:10.1371/journal.pone.0246716)

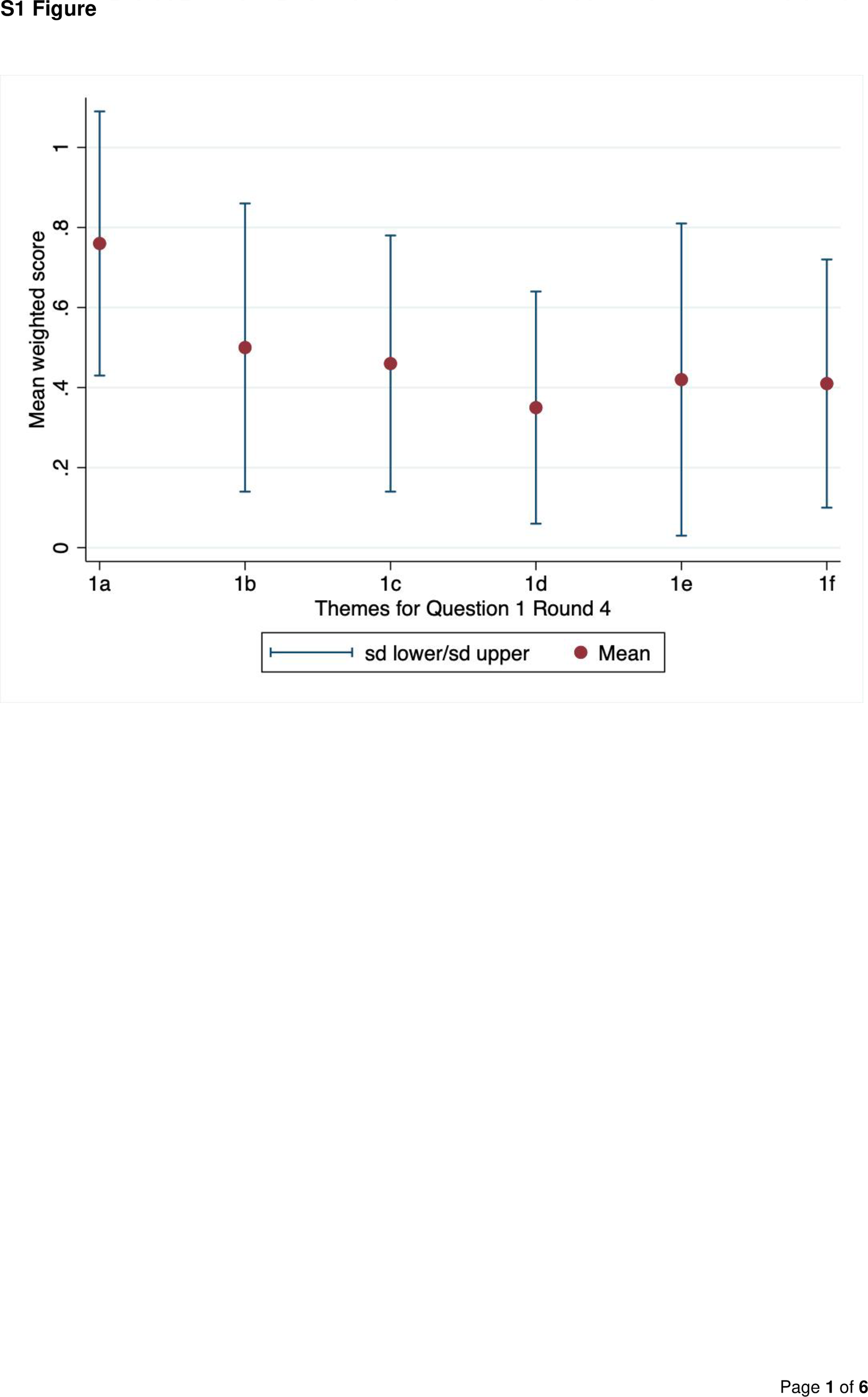

Supplement: S1 Fig — (TIF) [file pone.0246716.s001.tif]

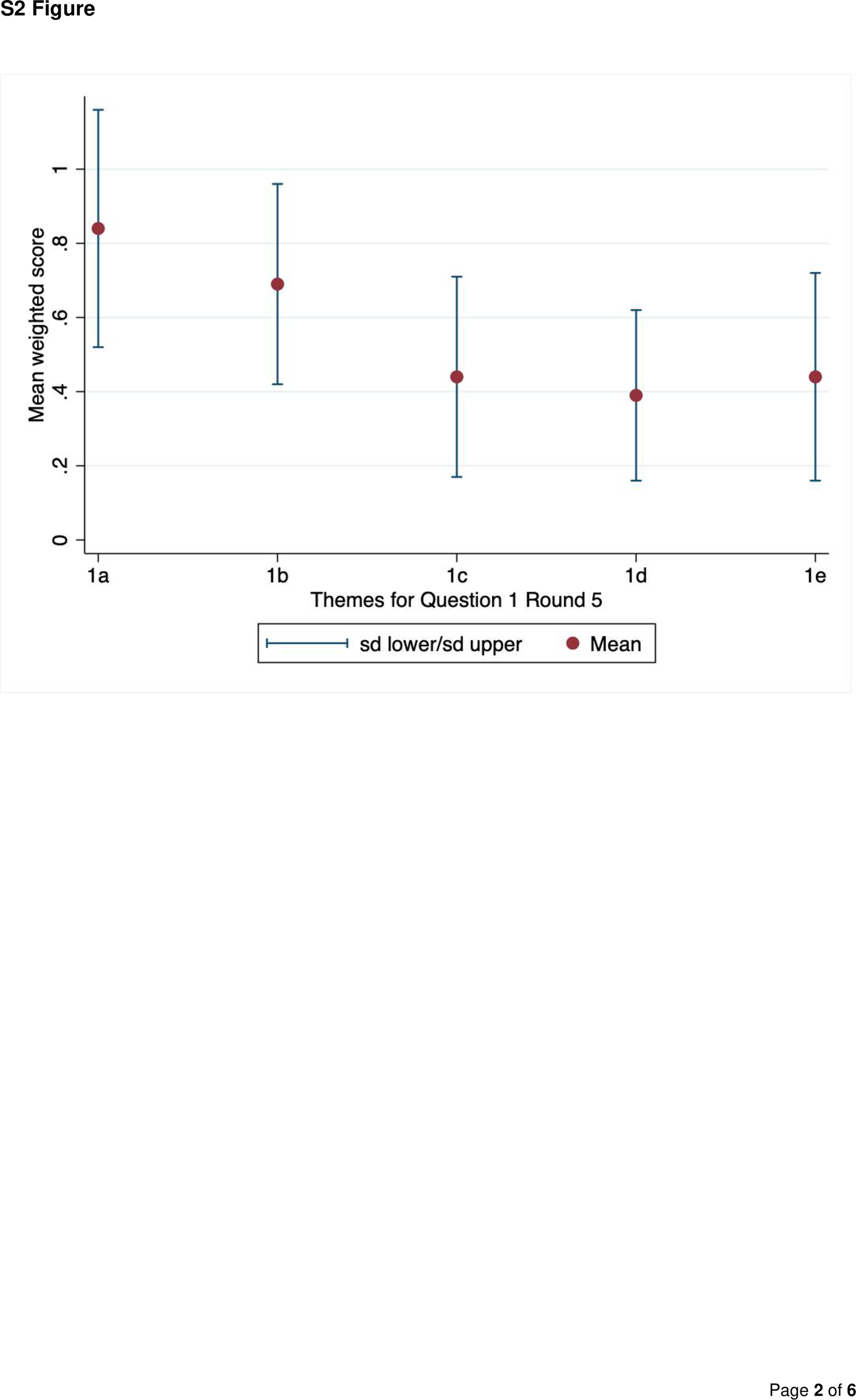

Supplement: S2 Fig — (TIF) [file pone.0246716.s002.tif]

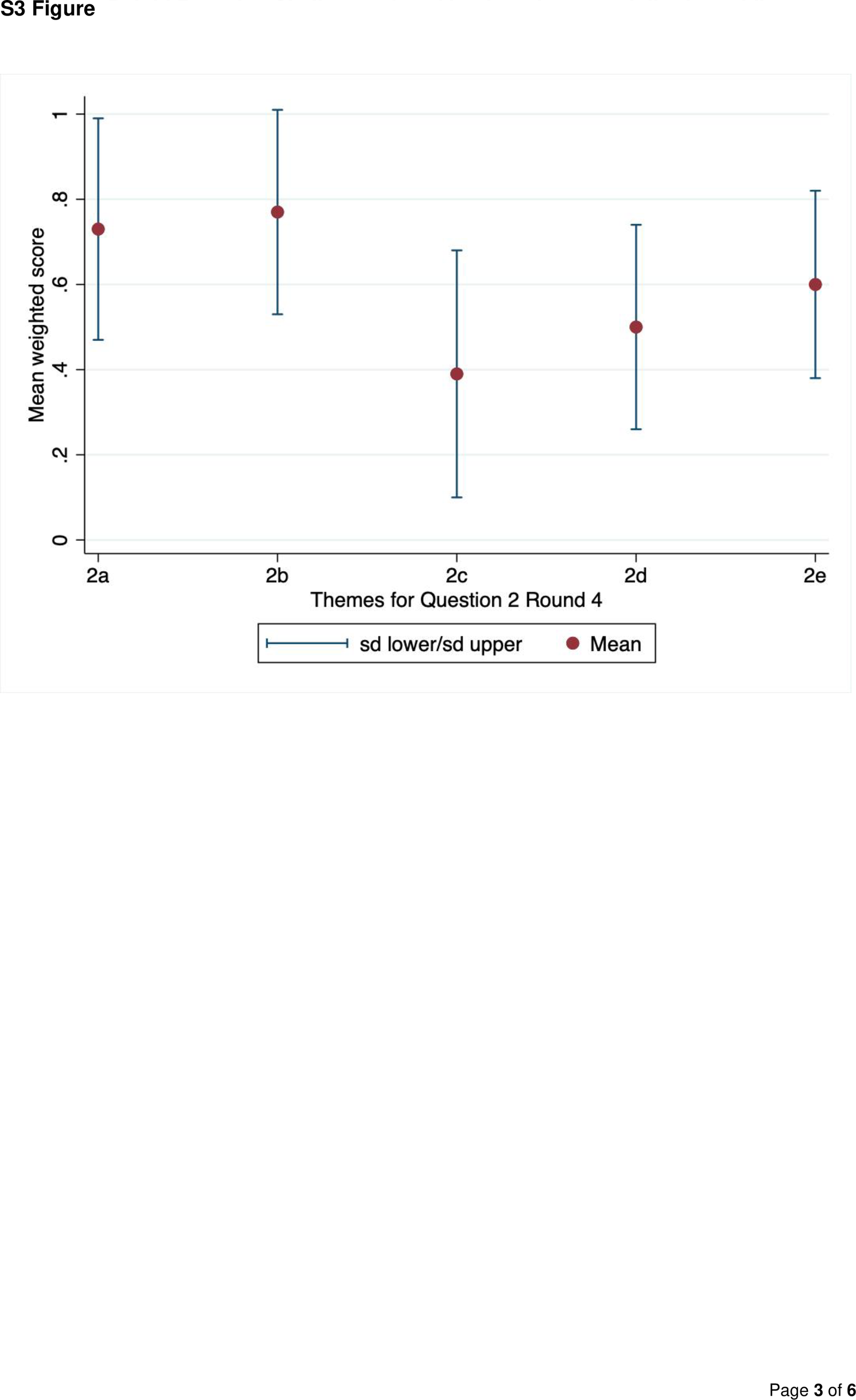

Supplement: S3 Fig — (TIF) [file pone.0246716.s003.tif]

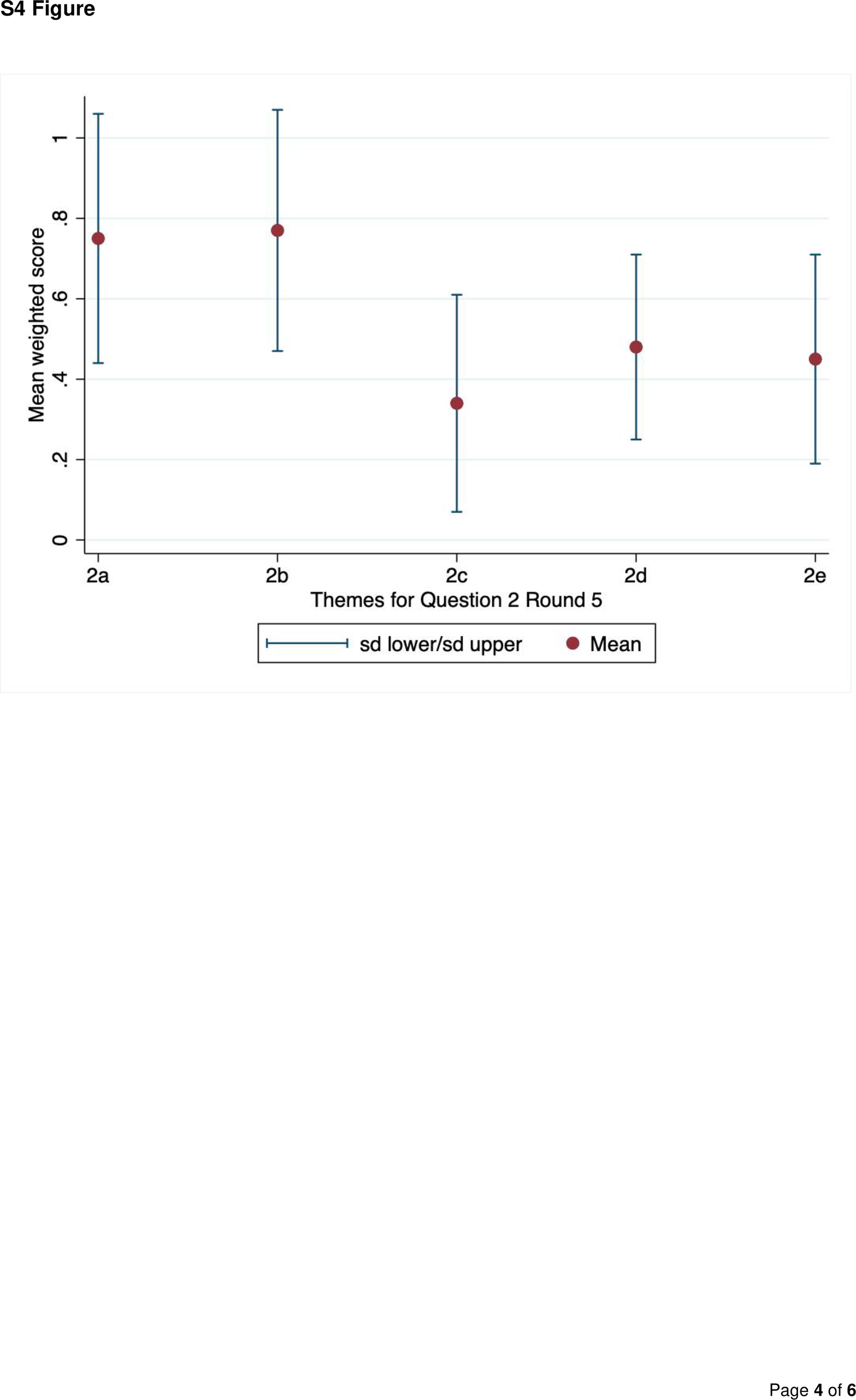

Supplement: S4 Fig — (TIF) [file pone.0246716.s004.tif]

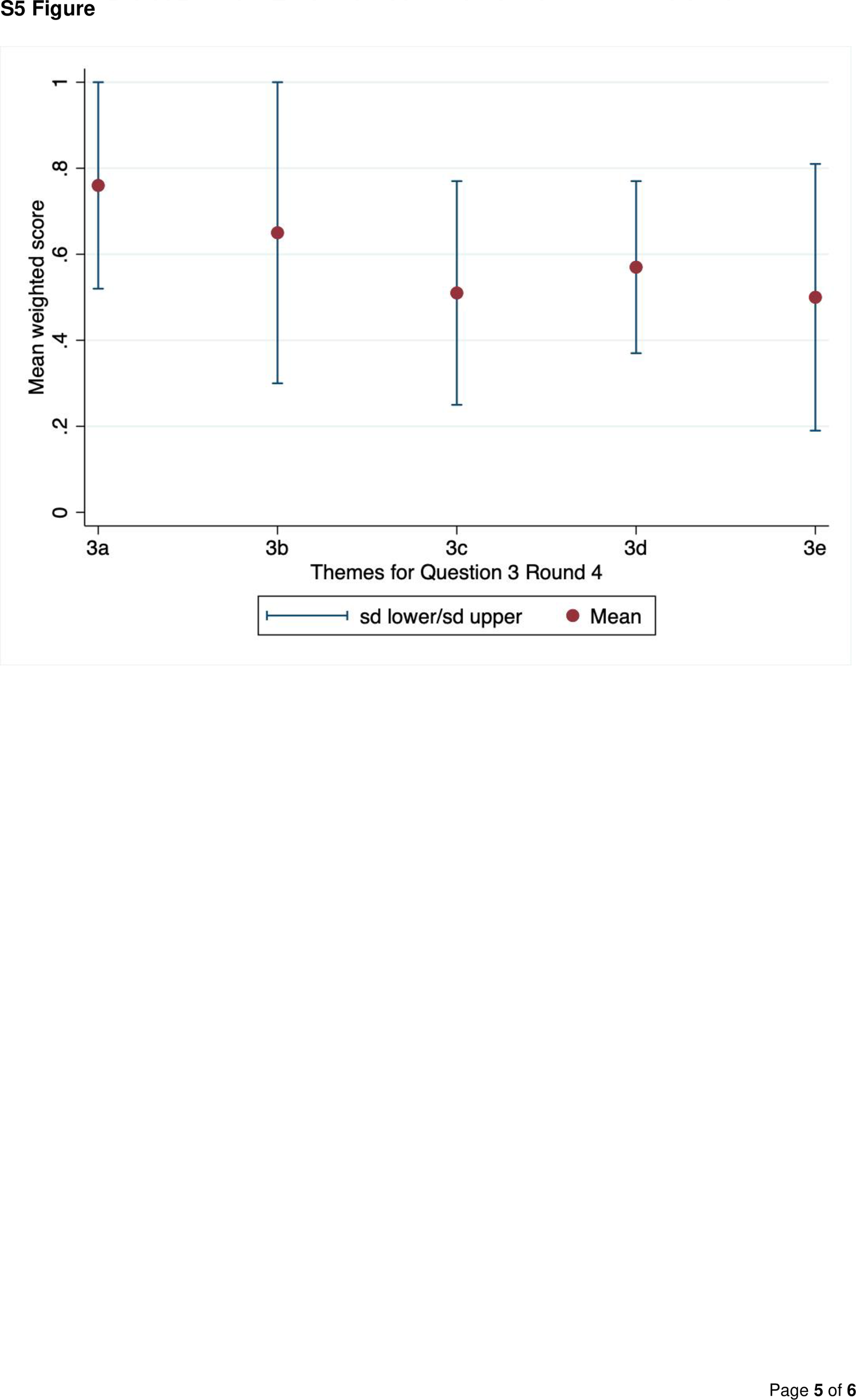

Supplement: S5 Fig — (TIF) [file pone.0246716.s005.tif]

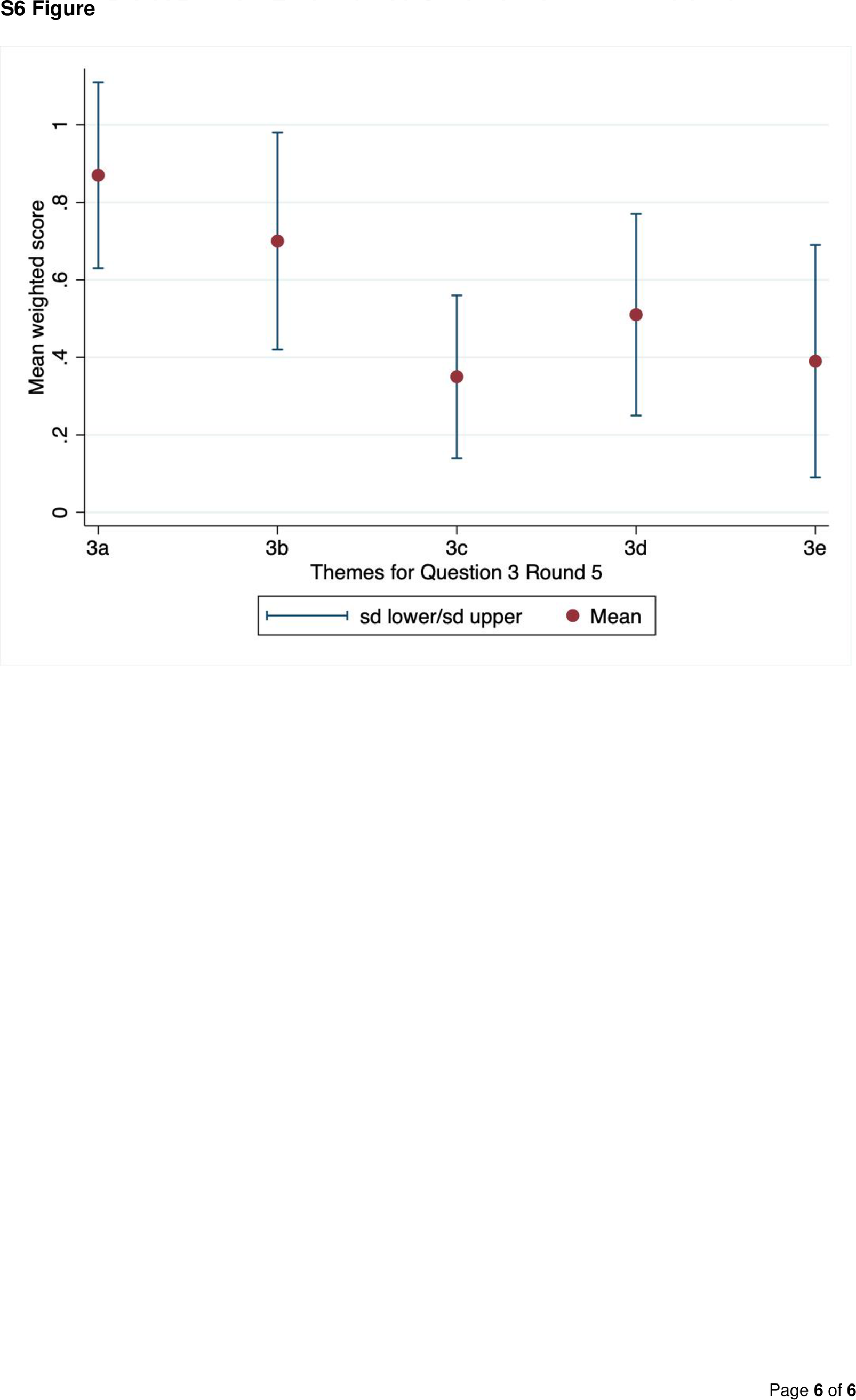

Supplement: S6 Fig — (TIF) [file pone.0246716.s006.tif]
